# A Review of Exoskeletons Considering Nurses

**DOI:** 10.3390/s22187035

**Published:** 2022-09-17

**Authors:** Esther Rayssiguie, Mustafa Suphi Erden

**Affiliations:** School of Engineering and Physical Sciences, Heriot-Watt University, Edinburgh EH14 4AS, UK

**Keywords:** exoskeleton, robotic assistance, medical robotics, design, nurse, healthcare, hospital, care home

## Abstract

Daily tasks of nurses include manual handling to assist patients. Repetitive manual handling leads to high risk of injuries due to the loads on nurses’ bodies. Nurses, in hospitals and care homes, can benefit from the advances in exoskeleton technology assisting their manual handling tasks. There are already exoskeletons both in the market and in the research area made to assist physical workers to handle heavy loads. However, those exoskeletons are mostly designed for men, as most physical workers are men, whereas most nurses are women. In the case of nurses, they handle patients, a more delicate task than handling objects, and any such device used by nurses should easily be disinfected. In this study, the needs of nurses are examined, and a review of the state-of-the-art exoskeletons is conducted from the perspective of to what extent the existing technologies address the needs of nurses. Possible solutions and technologies and particularly the needs that have not been addressed by the existing technologies are discussed.

## 1. Introduction

Daily tasks for nurses include a lot of repetitive manual handling and lifting when assisting the patients. Those tasks require a large physical effort and lead to heavy loads being applied on nurses’ body, resulting in high musculoskeletal injury rates. Manual lifting and transferring of patients, which places stress in the ligaments of the spine, was found to be one of the main reasons for musculoskeletal injuries with nurses [1,2,3,4]. The most prone area to get injured for nurses is the lumbar spine [4,5,6] but injuring their neck, shoulder and knees is also very common. In 2013, the National Health Survey (NHS) in the UK reported that, 6000 staff missed work every day because of musculoskeletal problems [7]. In addition, the UK population is getting heavier and older, more of them being obese [8,9], and obese subjects being more represented in hospitals [10,11], which implies the increasing significance of the problem with the manual handling tasks of nurses.

Manual handling of a greater number of overweight patients applies an even greater load on nurses. It is recommended for nurses to use equipment instead of manual handling when servicing patients [11,12,13]. To handle patients, a wide range of equipment is available to nurses, including: hoists (to raise patients from the floor, standing, mobile and bath hoists), lifting slings, slide sheets, transfer boards, stand aids, turntables, electric profiling beds, handling belts, bariatric equipment [14]. However, researchers found that nurses do not always use the available equipment when needed. This is due to different factors, such as time, complexity of the task, and equipment issues [2,15]. Nurses would perform the handling task manually in a critical situation as it is faster than using equipment [2]. Issues with equipment further make it less likely for nurses to use them as their size (do not fit into the room) and shape (too small for patient) might not be suitable for many servicing tasks. Furthermore, lack of equipment in hospital is also noted as an obstacle for nurses to get used to using them [2,15]. Even when using equipment, large loads are still being applied on nurses’ spine [3,6,16].

Exoskeletons could assist nurses by supporting them and enhancing their strength for manual handling as well as decreasing the physical effort and lowering the risk of musculoskeletal injury. There are already exoskeletons in the market and in research and development made to assist physical workers to handle heavy loads. However, most exoskeletons are designed for industrial physical workers, who have different type of tasks and the majority of whom are men. Nurses work in hospitals, meaning that they need to follow cleanliness guidelines (that would apply to the exoskeleton as well), safety measures to work close to patients (the exoskeleton needs to be safe to the user as well as to the patient) and handling guidelines. Handling a patient is more delicate than handling an object; this implies that the design of an exoskeleton for nurses would need to consider close contact with patients. Characteristics, such as having low pressure points between the human and exoskeleton, allowing all day use, and covering sharp edges, electric cables, motors, and joints would need to be implemented on an exoskeleton for practicality and safety. Nurses’ tasks involve specific body postures and motions, such as twisting and lateral bending which are difficult to achieve with a conventional rigid exoskeleton structure [17,18]. Moreover, most nurses are women [19]: women have a different anthropometry, body shape, and strength compared to men [10,20]. Those issues and factors specific to nurses are usually not addressed by the currently available exoskeletons [21].

In this study, we have reviewed the literature on nurses’ manual activities and the exoskeletons in the market and research. We used the key words nurses, activities, manual handling, musculoskeletal injuries to find out the literature on nurses, and the key words nurses, exoskeleton, soft, passive, powered, load, upper-body, lower-body, knee/back/shoulder/neck support to find relevant exoskeletons, technologies, and research. We searched in Research Gate, Science Direct, IEEE Xplore, Web of Science, Semantic Scholar, and National Library of Medicine databases for academic literature and performed Google search to find the technologies and other sources available online. We have reviewed the literature considering the aspects of comfort for women, controlled support, and mechanical features. Based on these aspects, we constructed a matrix of the most relevant papers, Table 5 in Section 5, to provide a single shot and concise summary of our review and we suggest the reader to refer to this matrix while reading the paper.

In the next section, nurses’ activities, and injuries due to the manual handling of patients are discussed. In Section 4, the needs of nurses and the challenges of manual handling are presented. Section 5 presents a review of relevant exoskeletons to analyze the possible solutions and technologies in existent work to address the needs and challenges of nurses in manual handling. Its purpose is also to identify the gaps in research and technology to develop an exoskeleton for nurses. Section 6 introduces a preliminary conceptual design of an exoskeleton for nurses based on the discussions in light of the review and discussions throughout the paper. The conclusions are then presented in the last section.

## 2. Nurses’ Activities and Injuries Due to Specific Movements during Manual Handling of Patients

In this review, we focus on the services of nurses that require physical force, specifically while servicing patients. This concerns nurses in hospitals as well as in care homes. In Table 1, the activities performed by nurses for servicing patients and demanding physical effort are identified, from a list of their activities given by the NHS [22].

The tasks which put nurses the most at risk, apart from lifting a patient, include pulling a patient in bed and transferring a patient from bed to stretcher or bed to chair [23]. NHS reported that 6000 NHS staff miss work every day because of musculoskeletal problems [24]. Potential high risk for musculoskeletal injuries associated with patient handling tasks include: high force (overexertion), transfer distances, confined environments, variable patient behaviour, awkward postures (stooping, bending and reaching), and repeated activities (lifting, transferring and re-positioning) [25]. The most prone area to get injured for nurses is the lower back [26,27,28,29] but injuring their neck, shoulders, wrist and knees [1,30,31,32,33] is also very common. An investigation [34] of the prevalence of musculoskeletal injuries, in a sample of 1163 nurses working in the United States, found that 47% had experienced back injuries within the past year. The risk of getting injured is accentuated with repetition.

### Why Are Good Handling Techniques Not Sufficient to Avoid Injuries?

Even if lifting is performed correctly, the process places a great deal of strain on carers’ bodies. The NHS has guidelines on how to perform manual handling [24]. However, following these guidelines is not sufficient to prevent lift-related injuries. Lifting and moving patients manually still place a great deal of pressure on the spine, regardless of the technique used [3,28,35,36].

Studies [3,36] found that the lifting process itself contains several inherent risks that lead to injury, due to:

1. Distance: It is much easier to lift something if it is very close to the body. However, the environment (bed, chair) can get in the way. Thus, nurses cannot get close to patients.
2. Bending: The majority of the force goes from bones along the spine directly to disks in the back, straining them.
3. Repetition: Each time a nurse lifts and moves a patient, there’s a risk of developing small tears inside disks in the back.

**Figure 1:**
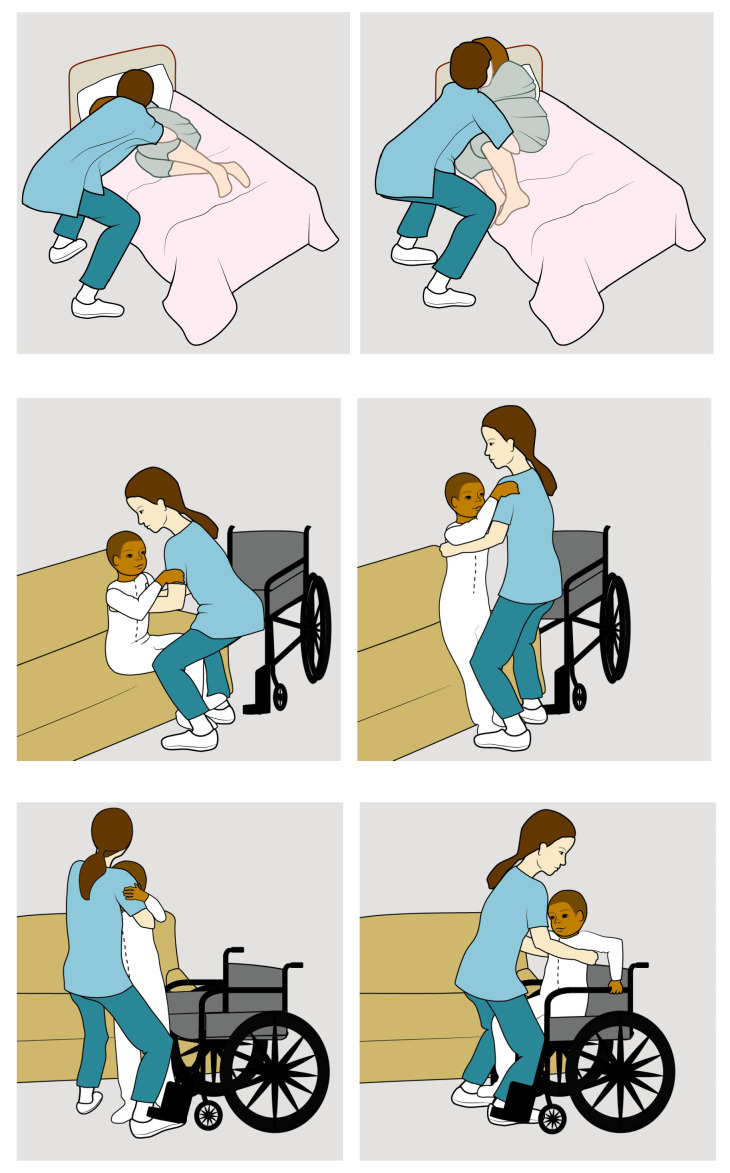
Assisting standing up in a bed, standing up from and sitting down in a wheel chair, requiring the career to bend at a distance [39]. Reproduced with permission from OrthoInfo. © American Academy of Orthopaedic Surgeons. https://orthoinfo.org/.

Those risks are present in many daily tasks that nurses perform, such as bed transfer, requiring nurses to bend and lift a patient at a distance [37]; assisting standing up from a chair, requiring the nurse to bend [38]; assisting standing up in a bed, standing up from and sitting down in a wheel chair, requiring the carer to twist and bend at a distance (Figure 1) [39].

In addition, lifting a patient can be unpredictable and is different from lifting an object of the same weight. Patients can be combative, can resist being lifted, have sudden muscle spasms, and have a moving center of gravity around multiple pivot points. Each of these reactions can create greater loads acting on the carer’s spine compared to when the lift is performed smoothly and well controlled, with the person being lifted remaining still.

Patient handling tasks, such as transfer from a bed to a chair (Table 1), require nurses to lift the patient while twisting. Twisting movements of the spine expose soft tissue to compression, sheer and strain forces. Twisting when lifting, lowering, or carrying any load increases the risk of back and neck injury. This is because the inter-vertebral discs in the spine do not handle shear force as well as compression force. Nurses can team up to reduce the weight each person has to handle when lifting a patient, and it can reduce compression forces on their spines, but teaming up is not as such effective to reduce the shear force. Two-person transfers still result in great loads applied on the spine. The study [35] identified that roughly 15% to 20% of the two-person transfers resulted in compression forces above the 6400 N tolerance limit. Patient transfers have been found to be the task that is most associated with the lower-back injuries suffered by nursing aides [35].

Lifting patients multiple times a day places the carers at high risks to injure themselves. Tasks such as getting the patient out of a chair (Table 1) require leaning while lifting. This results in a lumbar extension moment from back muscles and ligaments forces acting at short moment arms about the spine. The muscle and ligament forces represent most of the loading experienced by the inter-vertebral discs during such a forward leaning. Lifting a weight also causes large forces on the knees and wrist and can result in musculoskeletal injury [25,33,40].

Stoop lifting is recognized as an improper technique for lifting, however, nurses sometimes do it. Compared to the proper lifting technique, with knees bent, stooping includes keeping knees straight. This motion applies a lot more forces on the spine due to the flexion moment being higher compared to the proper lifting technique.

Tasks such as adjusting the patient in bed (Table 1), require nurses to do a transfer at a distance. Those tasks require leaning over, forward, and lateral bending, that are designated as high-risk movements for the spine and the inter-vertebral discs. During those tasks, forces are experienced by the chest, knees, and lower back of the subject. The majority of the force goes from the bones along the spine directly to the disks in the back, applying strain on them. Lateral bending motion applies more load and shear force on the spine compared to forward bending. The combination of lateral shear and compression increase the risks of carers getting back and neck injuries.

As in many other developed countries, the UK population is getting older and with that, their mobility will become more compromised in the future. The population aged 65 years and over is growing faster than other age groups in the UK. In 2018, around one in every five people were 65 years and over (18.3%) [41]. This age group is more represented in hospitals and in addition [11] handling them requires nurses to be more careful as elderly are more delicate. Thus, the rate of elderly patients is increasing, making manual handling more complex, and applying greater loads on nurses’ spines [7].

Work-related musculoskeletal disorders and injuries among nursing staff are a major concern also due to the growing weight of the patient population. Nurses get injured due to frequent moving and lifting of patients, especially if the patients are obese (BMI > 30 kg/m2) or overweight (25 < BMI < 29.9). Obesity is a common problem in the UK: according to NHS reports, around one in every four adults, 25%, are obese and 62% are overweight [11]. In England, more than 30% of the people aged 65 years old and more are obese, and this number gets higher each year [10]. On a daily basis, nurses are consistently handling more and more overweight and obese patients [11,42]. Bariatric patients are more difficult to handle and require more re-positioning to avoid medical emergencies such as respiratory distress, impaired circulation, nerve damage, and cardiopulmonary decompensation [43]. These patients should not be lifted manually, and carers should use equipment [12,25]. However, the amount of equipment for obese people is limited and/or cannot be used (due to confined spaces/lack of space, not knowing how to use it [44], stored elsewhere because too large) and the carers are not all trained to know how to use this equipment. Bariatric patients require a greater number of staff to assist them. However, in many busy hospitals, there are not enough staff members available to mobilize a lift team whenever a patient needs assistance. The study [45] showed that there is a significant relationship between wrist and knee pain and the number of manual handling tasks completed per hour of interacting with the load being lifted.

To design an acceptable exoskeleton for nurses, one needs to be aware of the anthropometry and size of nurses, especially in contrast to the male workers for whom most of the current exoskeletons have been designed. Ideally, an exoskeleton should be adjustable to the size of each user. However, making an exoskeleton adjustable for every body size would result in a heavier and more complex design, comprising the needs of nurses for daily use. Therefore, a practical solution might be to design exoskeletons for a majority of nurses, for whom the range of body size needs to be identified. This solution would reduce the weight of the exoskeleton and make the system easier and more practical for everyday use in hospital and care home environments. Nursing jobs are mostly occupied by women, 89.3% of nurses and midwifery are female [19]. The most common age profile of nurses varies between 25 and 54 years old [19]. For this age population, in Scotland, women’s height varies between 161.5 and 163.9 cm [10].

The anthropometry study [46] measured the proportions of the male and female body to obtain standardized proportions of the human body from their height. Using the nurses’ average size, we can get the height of most nurses and use those values to find out nurses’ proportions based on the study [46]. The measurements of most woman nurses, specifically the measurements of women in Scotland with height in the range 161.5–163.9 cm, have been calculated and then rounded as shown in Table 2. Those values are useful to know how much the exoskeleton should be adjustable to fit most nurses in Scotland. The last row of Table 2 gives a range of measurements, for different part of the body, that an exoskeleton should be adjustable for. Following a similar method, body measurement ranges for nurses in other countries can also be calculated.

## 3. The Needs of Nurses to Be Addressed to Overcome the Challenges of Manual Handling

This section identifies criteria to be considered when designing an exoskeleton for nurses and explain why those criteria are important. The criteria are split into two main parts, comfort and mechanical aspects. Both aspects are to be taken into account to develop an exoskeleton useful for and acceptable by nurses. The review of the existing exoskeletons in Section 5 will be made based on those criteria to critically compare the exoskeletons from the perspectives of addressing the needs of nurses.

### 3.1. Comfort, Size, Body Shape, Cleanliness

To be widely used and to assist nurses all day, comfort is an important aspect for an exoskeleton. Comfort is still challenging to achieve when the exoskeleton has to redistribute forces to the user’s body [47,48,49]. Analysis of the pressure applied by an exoskeleton showed that lowering or redistributing the pressure on the human body increases comfort and the acceptance of the device [50,51,52]. The pressure points and contact between the user and the exoskeleton should be well thought to avoid any discomfort and possible injuries [53,54,55,56].

The exoskeleton should be adjustable to fit a wide range of nurses, as seen in Section 3.1. It could be custom made to each user but then the price would be too high. The exoskeleton should have adjustable size of attachment to match the user’s size. Exoskeletons have been shown to have a better efficiency of assistance when they fit well to the user [18,57,58,59]. To fit well to the user, the measurements and the proportion of the human body must be considered.

Only a few exoskeletons for heavy lifting are made adjustable for an average woman [58], as most of them are designed for physical workers, who are mostly men. Most of the time, the average shape and dimension of women’s body is extremely different from the average shape of men’s body. They also have different friction points and points of sensitivity to consider for dynamic movements. Furthermore, nurses are rarely involved in the design of exoskeletons which could benefit the use and acceptance of exoskeletons in healthcare.

Nurses have to respect high standards of cleanliness followed in hospitals. As any equipment, an exoskeleton for nurses also has to be regularly cleaned and disinfected. Any fabric part of the exoskeleton should be detachable to be washed and the rigid parts including the actuators should be covered or removable to enable easy disinfection.

The exoskeleton should be able to support nurses throughout their working time. Passive exoskeletons can be used all day, apart from some of them that apply too much pressure on the user which can be uncomfortable. However, most powered exoskeletons need a battery and cannot be used for a long time. The exoskeleton for nurses must be lightweight, else too much stress would be applied on the user’s body, making it unlikely to be used often. The exoskeleton must also be portable, so nurses are able to help patients in different areas of the hospital without restriction.

Some exoskeletons provide an adjustable level of assistance [50,58]. The user can choose which level is needed depending on their task or the weight to be lifted. Having constantly a high level of assistance can be uncomfortable. For example, by using strong springs or elastic bands, the exoskeleton might apply a lot of pressure on the user and might become painful in time. The level of assistance should be adjustable either automatically by using sensors or manually by the user.

### 3.2. Mechanical Aspects: Kinematics, Power, Simple Design

As discussed previously, nurses’ activities involve twisting, bending, forward leaning, and lateral leaning [22]. Therefore, the exoskeleton should offer kinematic compatibility to allow those motions. If not, the device could be seen as bothersome [18,60]. Exoskeletons with a rigid structure are more likely to restrict the range of motion [17]. Materials that can bend and artificial joints allowing every or most motions should be focused on.

Nurses work closely to patients when doing manual handling tasks. By using an exoskeleton, they should still be able to get close to the patients to lift them. Therefore, the exoskeleton should not be bulky to complicate nurses’ tasks. Equipment is sometimes too big to fit in a small hospital room [12], but this should be avoided for the exoskeleton.

When handling a patient, their actions can be unpredictable, or they can be uncooperative. Therefore, the exoskeleton must not have wires and extensions that are not meant to be grabbed by the patient or have uncovered actuators. The structure needs to be as simple as possible and not harmful for the user or the patient. This issue is addressed by the exoskeletons specifically made for nurses but there are still too few of them [54,55,56,57,58,61]. 

## 4. Review of Exoskeletons

This section is a review of the existent exoskeletons to identify to what extent they address the discussed criteria to assist nurses. In this study, exoskeletons are grouped into three categories. Powered exoskeletons are made from rigid material to enhance the strength of the user. They are equipped with sensors and a power source to drive actuators (pneumatic, motors, hydraulic) [17,54,58,62,63]. Passive exoskeletons use mechanical actuation and/or combinations of springs and dampers to store energy from human motion and use it when needed to assist the user’s posture or motion [48,52,59]. They do not use a power source, and hence they are lighter than powered exoskeletons and present fewer safety risks to the users [64]. Soft exoskeletons are made from only or mostly soft material, such as textile. This results in them being extremely light, more compliant with the body and more flexible, allowing a wider range of motions for the user. On the contrary, soft materials result in a poor redistribution of forces on the user’s body due to not having a rigid frame. They might be powered and transmit power with flexible materials (such as Bowden cables, air muscles, filaments) [57,61,65,66]. We will review these three groups of exoskeletons from the perspective of comfort, size, body shape, cleanliness, support of the body, and adjustable support and will discuss to what extent they potentially fulfill the needs of nurses for these considerations. Our goal will be to identify the gaps and thus to find out what novelties are needed in exoskeletons to address the majority of the needs of nurses.

### 4.1. Comfort, Size, Body Shape, Cleanliness

Comfort is a key aspect for the design of the exoskeleton as it is critical for the users’ decision to wear it again or not. Currently, most exoskeletons are seen as uncomfortable and thus are not widely adopted [52,54,59,62]. Some are also viewed as too cumbersome to be used in hospitals during close contact with patients [67,68,69]. To be comfortable, an exoskeleton needs to have an adjustable size and joints that can rotate exactly around the same axis as the user’s joints.

**Figure 2:**
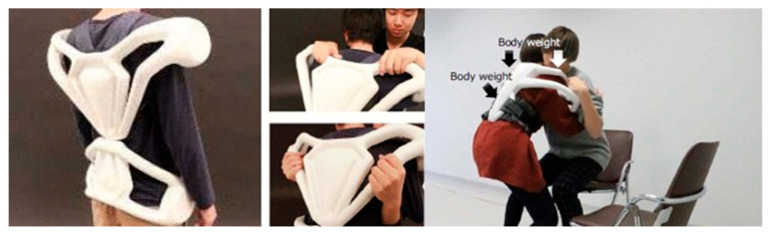
Hydraulic Exoskeleton for carers involved in assistance and transfer for bathing patients [54]. Reprinted with permission from author [54].

Exoskeletons re-distribute the forces exerted on the body to avoid the risk of musculoskeletal injuries. Some of them re-distribute the load on the back of the user towards the legs to avoid low-back pain [48,52,70]. An exoskeleton [54] designed for nurses to assist patients during transfer tasks was made to distribute through the frame to the user’s body the weight generated when the patient grips the frame, as seen in the Figure 2 below. This is to help the user carry out easily standing and transfer assistance without applying too much force on them.

In order to feel less pressure from the exoskeleton, a larger moment arm (distance between human joint and the centre of the structure) can be used to avoid shear forces to the user. With a large moment arm, the exoskeleton in the study [65] was designed to reduce the force applied to the limbs and the pressure on the limbs was reduced by using soft padding not to cause discomfort.

Being adjustable in size increases the range of users of an exoskeleton. For that purpose, different methods have been used. For a rigid structure, a slide system has been used in [17]. The VEX exoskeleton made the size of the back adjustable by up to 18 cm [50]. The attachment to the limbs of the user was made out of a Velcro belt or straps. This is often used in exoskeletons [17,58,62,70].

With joint misalignment, the exoskeleton becomes mechanically over constrained, causing excessive force at the attached location on the user. A solution proposed for this purpose is to use an algorithm that automatically adjusts the exoskeleton to the user’s body. The study [71] developed a system in order to align the mechanical knee joint to the user’s knee. A model and simulation of a human knee was made to analyze its motions. By using this simulation and by tracking the angle/velocity motions of the user in real time, the mechanical knee reproduced the motions of the real knee.

**Figure 3:**
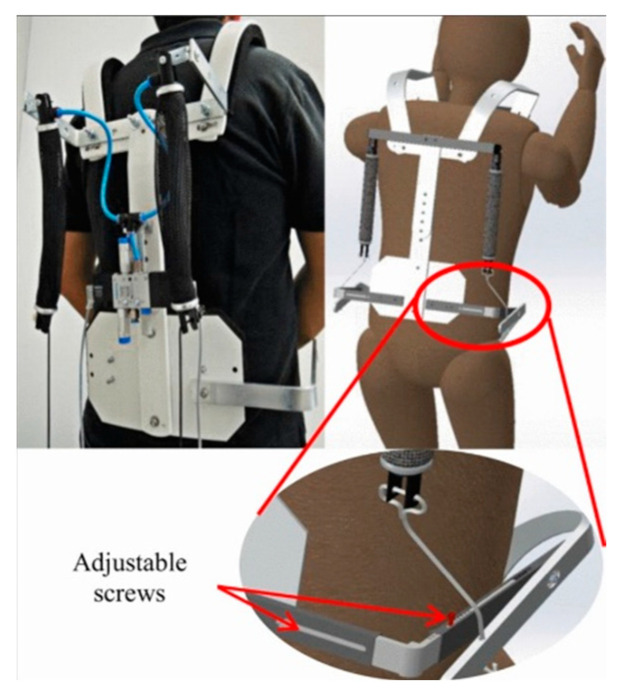
Backbone pneumatic exoskeleton [63]. Reprinted with permission from IEEE. License Number: 5383570454208; license date: 7 September 2022.

The particular exoskeleton shown in Figure 3 offers the possibility to interchange the place of some pieces and the dimensions of the mechanical structure. This allows the back structure, leg’s support, and shoulder’s support to be modified. In this way, the exoskeleton is adaptable depending on the user’s size and preferences [63]. This is an interesting solution that is not widely used yet.

**Figure 4:**
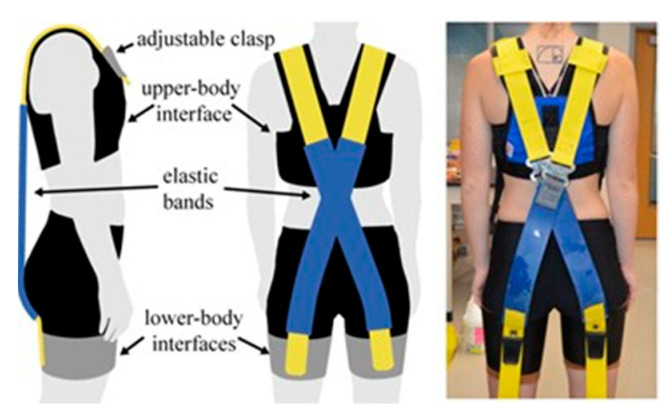
Bio-mechanically-assistive garment prototype [57]. Reprinted with permission from IEEE. License Number: 5383600890958; license date: 7 September 2022.

Only a few exoskeletons have been developed with consideration of women’s size and shape. Those are mostly soft exoskeletons [57] (Figure 4) [61,66,72] and only one of them is powered [58]. Those exoskeletons [57,58,61,66,72] are suitable for women because they are compliant to women’s body shape. They are designed considering sensitive areas of woman’s body (such as not having tight and rigid material on the chest, or having straight rigid structure on the hips), and redistribution of the pressure on the body. Rigid back-assist exoskeletons are usually not made for women. This is due to their chest plates, rigid structures lateral to the torso, and design around the hips and thighs [48,52,69,70,73] not being suitable for general woman body shape. Those features apply pressure on sensitive areas of women, making the device uncomfortable and difficult to move while wearing. Exoskeletons with hip belt usually do not have pad far enough, making it uncomfortable on the hips for women [74]. Another overlooked issue related to exoskeletons with hip joints and thigh plates is that they only allow movement in one direction. This encourages the device to shift towards the outside of the thighs of women. An option to overcome those issues could be to include adjustable hip joints, longer straps across the chest, adjustable artificial spine for different torso length, and better padding in certain areas to improve comfort. To fit the body well, exoskeletons covering the back can take the shape of the human spine [54,60,75]. By doing this, it can reduce multiple types of forces along the human spine such as the spinae muscle force, shear, and compression force of the lumbar vertebrae [75].

Some passive exoskeletons only have a frame structure and no actuators, making them easy to disinfect [48,52]. Soft exoskeletons mostly have their actuators detachable from the soft material. Elastic bands, fixed by clips, can be removed, as shown in Figure 4, or motors can be detached [66].

Exoskeletons can be relatively heavy (more than 5 kg), especially powered exoskeletons. This can be a burden on the user if they need to use the device throughout the day [62]. For such heavy exoskeletons, the weight of the exoskeleton and the weight of the carried load should be transferred directly to the floor [17,18,58,76,77]. Thus, the user does not endure those loads. However, with soft and passive exoskeletons, this is not a common approach as they are usually not in contact with the ground.

Exoskeletons actuated with motors or other actuators needing a power source have a limited use time. The exoskeleton usually has a battery that can be fixed to it, meaning that while charging, the exoskeleton cannot be used, or the battery should be removed. In the latter case, the user can use another battery while the first one is being charged [58,77]. The battery life, of the reviewed powered exoskeletons varies between 10 min [17] and 8 h of usage [58,77,78].

An issue with the current exoskeletons is that they are usually task specific [79]. Nurses have many tasks to perform, such as lifting, carrying, holding, walking, twisting, and stair climbing. Because of being task-specific, the current exoskeletons would decrease the performance of the other tasks than they are designed for. A solution proposed to address this, is the introduction of programming with exoskeletons. Programmable tasks could be selected to support the user in different tasks by controlling the degrees of freedom of the exoskeleton selectively to fit to the human-like motion in the specific task [80].

Most exoskeletons focus on the support of the lower-back, as it is the most prone area to get injured [1,27]. Some exoskeletons provide a support specifically for the neck area, but this is more relevant for users working in overhead environment [50,81,82]. A common issue that arises with back exoskeletons is the applied pressure on different parts of the body that would not have been affected otherwise [79]. Full-body powered exoskeletons can assist the whole body during lifting tasks [17,58,77]. However, the current full body exoskeletons are too bulky and impractical to be used by nurses.

A controller mounted on a powered exoskeleton can be used to enable the user to choose which level of assistance is desired. Usually, different options of pre-programmed assistance can be selected [58,70]. If spring or elastic actuators are used, they can be swapped by the user depending on the desired level of assistance [50,57,59,81,82]. Otherwise, a clutch can disengage the torque that assist the user [18]. Some exoskeletons have a clutch system that can also adjust the level of assistance [60].

### 4.2. Mechanical Aspects: Kinematics, Power, Simple Design

**Figure 5:**
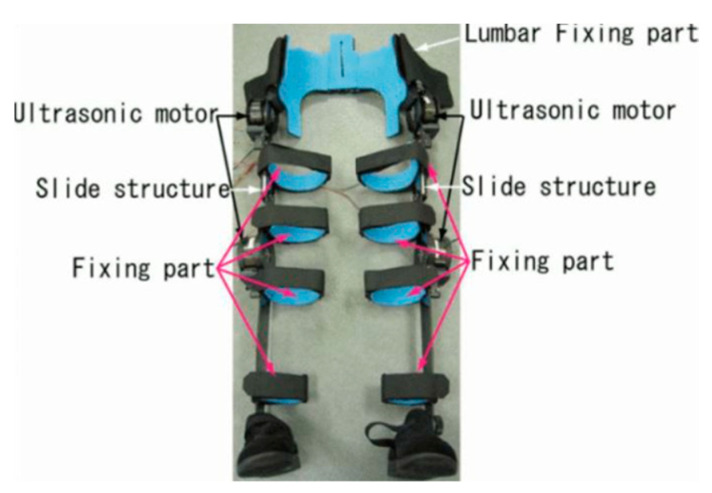
Ultrasonic Motor-Powered Assisted Suit System [17]. Reprinted with permission from IEEE. License Number: 5383601211803; license date: 7 September 2022.

Special attention has to be paid to nurses’ motions to ensure that the frame of the exoskeleton does not obstruct them during their tasks. This is challenging to achieve with powered exoskeletons as they usually have a rigid frame to enhance the strength of the user. Instead of using a metal structure, some powered exoskeletons were manufactured with engineered plastic [17], making twisting motions easier. To avoid restricting the user’s motions too much, the rigid structure can also have minimal contact with the user’s body. The full-body exoskeleton, shown in Figure 5, was made to only cover the user’s pelvis to avoid influencing the motion of the upper body and legs [17].

To align the joints of the exoskeleton and of the user, a slide structure can be used. As shown in Figure 5, a thigh slide structure was implemented to match rotational axis of the knee motor with the user’s knee. The exoskeleton in the study [50] uses a polycentric shoulder joint that provides multiple pivot points with 4-link assistance. This enables the user to have all of their motions. Unactuated joints can be implemented to allow unrestricted movements [70]. Mechanical joints can have passive degrees of freedom (DOF) and this helps in saving weight and power. Passive degrees of freedom mean that the exoskeleton allows motion in that degree of freedom, but the wearer will need to provide the required effort for the motion while handling the object [76].

Motors can be accurately controlled making it possible to adjust the level of assistance. The HAL exoskeletons [17,58] enhance the user’s ability to stand and walk by amplifying their own joint torque. Pneumatic actuators are simple to use, install, and maintain [63]. How- ever, they are less accurate and more difficult to control compared to electric motors. Hydraulic actuators can provide a lot of power, but they are more complex to integrate to the design and they can be difficult to maintain as their fluid may leak. The exoskeleton in [54] uses a micro hydraulic actuator to reduce the burden on carers during forward tilting action, twisting action and lifting action. Series elastic actuators are relatively quick to respond and energy efficient [18]. They have an elastic element with fixed stiffness placed in series with a motor. Series elastic parts do not require a power source and are often implemented in soft exoskeletons [57,61]. They are easy to use, to change and have a long-life cycle. Studies using the Smart Suit Lite [61] proved that by increasing the pressure surrounding the pelvis, the elastic belt on the torso increases the lumbar support, which in turn reduces the inter-vertebral discs pressure and stabilizes the posture. However, it is sometimes perceived as not providing enough physical support [67]. Springs do not require a power source, are simple to use, low-cost, and easy to maintain. They can be exchanged to give an adjustable level of support to the user. Springs proved to reduce muscles activity of the user and thus are also effective to reduce the risk of musculoskeletal injuries [50,59,60,73,81,82].

An exoskeleton for nurses needs not to be bulky and its actuators and cables need to be hidden to be safe to use in the vicinity of patients. Powered and rigid exoskeletons designed for general purpose [77] are usually too bulky to be used by nurses as they would make the lifting of a patient from a bed too difficult for example. On the other hand, soft exoskeletons are worn close to the body, can be worn underclothes and are safe to use around patients. The Aura suit [66] uses pods to hide all electronics and actuators. There is little chance for the patient to damage the suit while holding onto the nurse wearing it.

### 4.3. Comparison of Available Exoskeletons

In order to make a high-level comparison of available exoskeletons, we have grouped them into the three aforementioned categories in Table 3, where the advantages and disadvantages of each group have been identified. This comparison was made to identify which type of exoskeleton is the most appropriate and what aspects of each type might be useful in the design of an exoskeleton for nurses. The main points to conclude from Table 3 are that: powered-rigid exoskeletons can provide the most support to lift weight, passive exoskeletons can be used all day, and soft exoskeletons are lower-cost and compliant with the body. Each type addresses different criteria that were previously listed, in Section 4. An idea is to use different parts of each type of exoskeleton identified as useful for nurses, such as designing a powered-rigid mechanism to support some critical joint and mount this on a soft structure that complies with the body and provides passive assistance to other muscles and joint. Bringing the advantageous aspects of these different groups of exoskeletons together in a single design is a challenge as the working principles and actuation mechanisms might not be compatible with each other and the overall physical design might not be balanced for physical sustainability and considering the fitting to human body. However, as far as our study indicates, addressing these challenges might be possible and promising to develop a practical and desirable exoskeleton for nurses.

In this section, we provided high level consideration of categories of exoskeletons. Table 4 shows a summary of the most relevant individual exoskeletons in order to provide a more detailed view of technologies and aspects that might be useful for designing an exoskeleton for nurses. The comparison table highlights the solution features of the exoskeletons that address the main concerns related to an exoskeleton for nurses.

Few of the exoskeletons reviewed [17,54,57,61,83] consider twisting or lateral motions, which is often done by nurses during manual handling tasks. Just a couple of them [17,52,54] paid particular attention to the safety and comfort of the patient. From Table 4, we identified six groups of available exoskeletons considering that they are relatively similar and that they address similar issues. Using these six groups, Table 5 was made to deduce the best solution to design an exoskeleton to support nurses and assist them in their manual handling tasks. It shows the criteria, listed in Section 4, met by the exoskeletons reviewed. A few exoskeletons had to be left out as they were only in the early prototype phase and/or there was not enough information about them. A check mark is given if the criteria are addressed. 

In Table 5, we observe that the soft, passive and powered exoskeletons address different kind of criteria. The groups that address most of the criteria are soft exoskeletons, commercialized spring actuated passive exoskeletons, and commercialized full body powered exoskeletons. Soft exoskeletons, and most passive exoskeletons, do not interfere with daily activities or with the motions of the user, and are also lighter. A simple design with fewer components is relatively low cost (Groups 1 and 3). On the other hand, powered exoskeletons provide more support when handling heavy loads, reducing forces applied on the user. Some of those exoskeletons use sensors to activate the actuators when needed and assist the user considering the muscle activity measurements, which increase their efficiency. A major issue with full body powered exoskeletons is their weight. The heavier ones support themselves but some of them, up to 12 kg, do not. Criteria that are more specific to nurses, such as the device having to be washable and lightweight for all day use, are only addressed by a few exoskeletons [52,57,61,66].

### 4.4. Recap

The three groups of exoskeletons address different criteria, listed in Section 4, for nurses. A lot of exoskeletons are still at an early stage of development, with many concepts not tested beyond the laboratory. Powered-rigid commercialized exoskeletons enhance the strength of the user to help them carry loads while limiting the risk of getting injured from lifting those loads. Lower body, trunk and upper body regions could benefit from large reductions in loading. They are efficient for physical workers, but in our case, they are usually too bulky, heavy, and restrict twisting motions (required for nurses’ activities). Passive industrial exoskeletons aim at sup- porting or unloading the lower-back and are efficient in both dynamic lifting and static holding activities. Concerns are raised regarding discomfort and not providing enough support for lifting heavy loads. Soft exoskeletons are compliant with the body and support the lower-back. Considering the activities of the nurses, their disadvantage is not to provide enough support when carrying loads.

Different individual solutions observed in the review can be effective in a hospital environment, such as hiding actuators in pods for the safety of the user and the patient. However, technical challenges, discomfort, and lack of adaptability for women appear to be significant challenges for implementation of these solutions. In the following section, as an outcome of this study, we present a preliminary conceptual design that might address these challenges and bring together the advantages of each group of exoskeletons in a single design. 

## 5. Most Important Characteristics and Preliminary Conceptual Design of an Exoskeleton for Nurses

Based on the identified needs and activities of nurses, and the review of available exoskeletons, the most important characteristics desirable for an exoskeleton for nurses would be:

- Design with a focus on women’s body shape,
- Pressure redistribution considering women’ sensitive areas,
- Patient friendly,
- Adjustable level of assistance,
- Allow twisting and bending motions,
- Lightweight and portable,
- Easy to wash and disinfect,
- Compact and intuitive system.

Based on the analysis of the literature review in the previous sections and the list of these most important characteristics, we propose a preliminary conceptual design of an exoskeleton for nurses.

**Figure 6:**
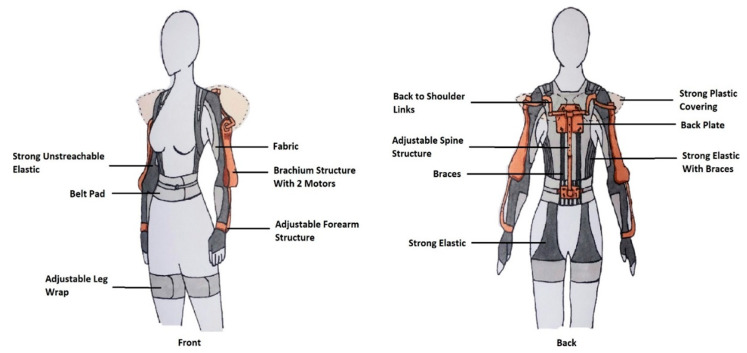
Sketch of the Conceptual Exoskeleton.

Based on the information in Table 5, in Section 5, an obvious solution for an exoskeleton for nurses would be to combine the powered-rigid exoskeletons, such as in Group 5, with the soft exoskeletons, such as in Group 1, and take advantage of both types. For the design of the proposed exoskeleton, special attention was given on how it would fit women, be comfortable to wear and be suitable to use around patients while supporting manual handling tasks. The proposed exoskeleton consists of a soft part and a mechanical part detachable form each other (Figure 6), and the powered-rigid part could be added when the nurse needs to lift a patient. The soft part covers the upper body and upper legs, and reduces muscle activity in the wrists, shoulders, and back. Strong elastics and braces, placed along the side and the back of the user, support the back during bending. A supportive belt reduces the loads on the lumbar spine. An elastic is attached up to the back and down to the wraps around the thighs to support bending and to reduce loads on the back of the user. The powered-rigid part covers the upper body and arms and includes electrical motors at the shoulder and elbow joints. The motors would help nurses carrying loads during lifting and transfer tasks. This would reduce the load applied on the lower-back and the spine by transferring it to the thighs and legs. The mechanical part is attached to the braces of the soft part. A male–female structure is installed to secure both parts together, and to easily detach the mechanical part from the soft part. The exoskeleton would have strong plastic covering to prevent access to the joints and electric components to ease the disinfecting of the device.

The exoskeleton’s shape and size were determined according to the proportion and measurement of women. For example, there is no chest plate included in the design and no rigid parts along the hips. The size of the exoskeleton is adjustable to fit the range of women nurses. The soft part of the exoskeleton can be made in different sizes and the mechanical part is adjustable for the identified range of size of women. For the soft part, the supportive belt is wide with padding along the side to be comfortable for women, as it was found to be a good solution in the review of the paper [75]. The belt is adjustable at the front. Straps are used to adjust to the size of the torso. Moreover, adjustable wraps, using Velcro, are placed on the thighs. These wraps are quite wide to avoid discomfort. For the mechanical part, slide structures were designed to fit users with different size. The size of each mechanical component, and their range of adjustment are based on the measurements calculated in Table 2. The assistance provided by the motors can be adjusted using sensorial feedback and motor control.

The exoskeleton was designed to be lightweight as its purpose is to be used all day. A battery is placed at the back of the exoskeleton to enable the device to be portable. The soft part of the exoskeleton does not weight much as it is mostly fabric and elastics. The mechanical part of the exoskeleton can be made from different materials. The adjustable spine structure at the back, and the links between the back and the shoulder can be made from carbon fibers as it has high stiffness, high tensile strength and is lightweight. For the arm components and the back plates, a hexagonal aluminum structure covered by fiberglass can be used. This type of structure is called a “honeycomb structure”. It is very strong and light as well. This would limit the use of carbon fibers and the exoskeleton would stay low-cost.

The design of the exoskeleton allows the user to detach the mechanical parts from the soft parts of the device. This design choice was made to allow the exoskeleton to be easily washed and disinfected. By covering all the electric components and the joints under a plastic structure, disinfecting becomes easy to perform. This preliminary design pays special attention to the needs of nurses and addresses the gaps identified in the review by bringing the advantageous features of different types of exoskeletons:

- Design with a focus on women
- Adjustable level of assistance to the arms with the actuators
- Patient friendly as the electric components are hidden and as there is no bulky structure on the front side of the user
- Lightweight and portable as a great part of it is made of light elastic material
- Allows twisting and bending motions
- Easy to wash and disinfect

## 6. Conclusions

This study reviewed the needs of nurses for manual handling of patients and the state-of-the-art technologies and research on exoskeletons, further highlighting the potential of an exoskeleton to assist nurses during their manual handling tasks, by enhancing their strength and reducing the risks of musculoskeletal injuries. There are issues with the existent exoskeletons from the point of view of being useful to nurses. In addition, only a few exoskeletons are designed for nurses, especially for women and for the healthcare environment.

We identified a wide range of criteria to be considered to assist nurses and identified potential solutions from academic papers and commercially available exoskeletons in order to address those needs. A challenge to develop a useful exoskeleton for nurses is to bring together these potential solutions in a single and holistic design. To that goal we made a fist attempt and based on our review we proposed a preliminary conceptual design of an effective and practical assistive exoskeleton for nurses.

The aim of the conceptual exoskeleton presented is to assist nurses during physical demanding tasks and reduce the loads applied on their body while carrying those tasks. Our review indicated that soft exoskeletons, made of elastic material, and powered exoskeletons, made of rigid material, both have advantages and address different needs of nurses. Mainly, whereas the soft exoskeletons are comfortable, are easy to clean, fit to the body, and allow flexible movements, the powered-rigid exoskeletons are advantageous to provide the required level of power assistance. Therefore, our review indicates bringing together the advantages of these two different systems in a single design. Accordingly, the proposed exoskeleton consists of a soft and mechanical part, working together to assist nurses. For the design of the preliminary-conceptual exoskeleton, particular attention was placed towards the comfort for women, the safety of patients, and the support and assistance to the user. Thus, the exoskeleton would be appropriate to use in a hospital setting.

This review paper focused on the physical structure and actuation of exoskeletons and did not discuss the sensors and machine intelligence that could be used to interlink the human body posture and human intentions with the high-level control of the exoskeleton. We note here that, estimating the body posture and intent of the user through sensors and machine intelligence embedded in the exoskeleton is an important aspect of exoskeleton design and development for both functionality and safety in human-machine interaction. Exoskeletons for nurses can specifically benefit from such technology as the tasks of nurses involve characteristically different body movements and configurations than those of other heavy load workers and as the exoskeleton is to be used in close vicinity to other humans. A consideration of advanced sensor technologies and identification of those most suitable to exoskeletons for nurses might deserve another focused review. We have been currently working on this and have succeeded in using IMU sensors to estimate the body posture adopted just before lifting a heavy load in order to initiate the assistance of the exoskeleton at that moment and to release the assistance once lifting is over. Machine learning techniques, such as deep learning, might be useful to interpret the sensorial data to estimate several body postures relevant to decision making for gradual assistance of the exoskeleton. Our future work has been progressing along these lines. 

## Figures and Tables

**Table 1:** Nurses’ activities for patients demanding physical effort.

Bedroom	Bathroom	Elsewhere
- Sitting up or moving up in bed	- Bathing	- Sitting in a chair
- Transfer from a bed to chair or vice versa	- Showering	- Standing
- Getting in or out of bed	- Using the toilet	- Walking
- Turning over in bed		- Getting up from the floor after a fall
		- Getting in and out of a vehicle

**Table 2:** Average measurements (in cm) of women nurses.

	Height (cm)	Back	Waist	Brachium	Forearm	Thigh	Shoulder
Women	161.5	48	35	31	24	17	39
Nurses	163.9	49	36	32	25	18	40

**Table 3:** Advantages and Disadvantages of Powered-Rigid, Passive and Soft Exoskeletons.

	Advantages	Disadvantages
**Powered-Rigid** **Exoskeletons** [17,18,57,58,62,63,70,77]	− Provide sufficient support when lifting weight − Automatically adjust to the level of assistance needed	− Energy driven (short endurance, large energy consumption) − Bulkier structure (poor environmental adaptability) − Solid structure (interferes with daily activities, reduce movements) − Not easy to maintain − Expensive
**Passive** **Exoskeletons** [48,50,54,69,81,82]	− Can be used all day − Easy to wash − Easy to use − Low-cost	− Do not provide as much support as powered exoskeletons
**Soft** **Exoskeletons**[57,61,66,68,83,84]	− Compliant with the body − Easy custom fitting − Smaller and lighter − Can be worn underneath PPE − Low-cost	− Motors and sensors difficult to mount − No rigid frame, less strength provided − Strain applied to the body

**Table 4:** Summary of the Reviewed Exoskeletons.

Reference	Targeted Users	Body Area Supported	Results Found from Experiments or Simulations	Suggested Solution by the Study
Naruse et al. [62]	Physical Workers	Lower-Back	Reduces upper body weight and muscle activity while bending and lifting weight.	Powered exoskeleton (exo) with cable and drum actuator.
PAS [17]	Nurses	Shoulders, Lower-Back, Knees	For transfer tasks.	Motor Powered exo made out of plastic for twisting motions.
HAL [58]	Nurses	Shoulders, Lower-Back, Hip, Knees	Reduces loads on spine during lifting of heavy weight. Increases user’s strength.	Motor Powered exo that supports hip flexion/extension and reduces trunk flexion.
Rosales et al. [63]	Physical Workers	Lower-Back	Reduces loads on spine during lifting of heavy weight. Increases user’s strength.	Powered exo using Pneumatic Artificial Muscles. Uses force sensors for adequate assistance.
Tashiro et al. [54]	Nurses	Lower-Back, Elbow joint	Reduces loads on nurses during bath-caring, involving standing and transfer assistance (titling, twisting, and lifting)	Powered exo using Hydraulic Actuators. Focus on patient for the design of the device (bars to grab for patient).
Zhang et al. [18]	Physical Workers	Lower-Back, Hip, Knees	Reduces lumbar spine compression during lifting of heavy objects.	Powered exo with motors and series-elastic actuators.
Mk2 [70]	Physical Workers	Lower-Back, Hip	Assist lower-back during lifting of heavy objects.	Powered exo with parallel-elastic actuator and unactuated joints.
Yu et al. [65]	Physical Workers	Knees	Assists squat and stoop lifting to avoid knee pain.	Powered exo using cable, high-torque motor, and large arm moment.
Yang et al. [75]	Physical Workers	Lower-Back	Assists squat and stoop lifting to reduce shear and compression forces on spine by 37% and 40% respectively.	Spine shaped soft powered exo using cable and motor.
CrayX [78]	Physical Workers	Lower-Back	Supports heavy lifting.	Motor Powered exo.
PLAD [59]	Physical Workers	Lower-Back	Supports user during bending. Lumbar muscle activity reduced by 14%.	Passive exo using Elastic springs system. Exchanges forces with the user at the spine, pelvis, and feet.
BNDR [48]	Physical Workers	Lower-Back	Reduces loads on spine during bending by 14%.	Passive exo using springs. Exchanges forces with the user at the chest, and thighs. Reduces torso flexion.
LAEVO [52]	Physical Workers, Nurses	Lower-Back	Reduces back muscles activity during bending by 35%.	Passive exo. Transfers loads from lower-back to chest and legs using tubes.
SPEXOR [60]	Nurses	Lower-Back	Reduces loads on spine. Assists bending.	Passive exo using springs. Clutch for level of assistance.
Han [73]	Physical Workers	Lower-Back	Assists during lifting and transfer tasks. Reduces loads on spine.	Passive exo using com- pression springs’ stored energy.
EVO [81]	Physical Workers	Neck, Lower-Back	Provides muscular assistance and prevent injuries.	Passive exo using springs. Different level of assistance.
Airframe [82]	Physical Workers	Shoulders, Neck, Upper-Back	Prevents musculoskeletal injuries. Reduces muscles activity.	Passive exo using springs. Different level of assistance.
VEX [50]	Physical Workers	Shoulders, Neck, Upper-Back	Assists during heavy lifting. Reduces muscle activity by 30%.	Passive exo using springs. Different level of assistance.
SSL [61]	Nurses	Lower-Back	Assists nurses during manual handling.	Soft exo using elastic belts.
Aura [64,66]	Elderly	Torso, Lower-Back, Hip, Legs	Assists during standing up and sitting down motions.	Soft exo using artificial muscles and sensors.
Lamers et al. [57]	Nurses	Lower-Back	Reduces loads on back. Reduces back muscles activity by 15% during lifting.	Soft exo using elastic bands. Redistributes forces.
Yu Z. et al. [83]	Healthy adults	Hip, Legs	Assists during walking. Decreases metabolic rate by 7.3% up to 14.6%.	Soft exo using Bowden cable, and elastics controlled by iterative learning control system.
Domenico C., et al. [84]	Physical Workers	Wrist, Hand	Assists during flexion of the hand. Reduces muscle fatigue and activity.	Soft exo using cable-driven actuator.
SIAT Soft Exosuit (SSEX) [85]	Healthy adults	Hip, Legs	Assists during walking by decreasing muscle activity.	Cable-driven soft exo with gait analysis.
Evelyn J.P, et al. [86]	Adults with difficulty walking	Knees	Assists knee extension when needed while walking.	Soft exo using some rigid components, straps, and Bowden cable.
Hee D.L., et al. [87]	Healthy adults, adults with knees difficulty	Knees	Supports knee joint to assist when ascending or descending stairs. Reduces muscles activity.	Soft exo with wire-driven actuator.

**Table 5:** Needs of nurses versus to what extent they are addressed by the available exoskeletons reviewed. The sign (✓) indicates that the need has been addressed by the corresponding set of literature.

	Type	Support Area for Manual Handling	Adjustable Size	Required Support Provided for Physical Tasks	Use of Sensors to Provide Effective Response	Portable	Washable/Disinfectable	All Day Use	Does Not Interfere	Easy to Put On	Weight	Adjustable Assistance	Easy Interface	Patient Friendly	Low Cost
Group 1: Soft Exo [57,61,66,68,83,84,85,86,87]	Soft	✓	✓			✓	✓	✓	✓	✓	✓	✓	✓	✓	✓
Group 2: Commercialized Spring Actuated Passive Exo [50,81,82]	Passive	✓	✓	✓		✓		✓	✓	✓	✓	✓	✓	✓	
Group 3: Passive Exoskeletons [48,54,69]	Passive					✓	✓	✓		✓	✓		✓	✓	✓
Group 4: Commercialized Full-Body Powered Exoskeletons [58,77]	Powered	✓	✓	✓	✓	✓	✓			✓		✓	✓	✓	
Group 5: Powered Exoskeletons for Lower-Back [51,63,78]	Powered	✓	✓	✓		✓		✓		✓	✓		✓	✓	
Group 6: Full-Body Powered Exoskeletons [17,18,62,70]	Powered	✓			✓	✓				✓			✓		
